# Mechanical Stimulus-Related Risk Signature Plays a Key Role in the Prognostic Nomogram For Endometrial Cancer

**DOI:** 10.3389/fonc.2021.753910

**Published:** 2021-10-06

**Authors:** Xin Xu, Xingchen Li, Jingyi Zhou, Jianliu Wang

**Affiliations:** ^1^ Department of Obstetrics and Gynecology, Peking University People’s Hospital, Beijing, China; ^2^ Peking University People’s Hospital, Beijing Key Laboratory of Female Pelvic Floor Disorders Diseases, Beijing, China

**Keywords:** endometrial cancer, mechanical stimulus, nomogram, risk model, overall survival

## Abstract

**Background:**

Tumor biomechanics correlates with the progression and prognosis of endometrial carcinoma (EC). The objective of this study is to construct a risk model using the mechanical stimulus-related genes in EC.

**Methods:**

We retrieved the transcriptome profiling and clinical data of EC from The Cancer Genome Atlas (TCGA) and Molecular Signatures Database (MSigDB). Differentially expressed mechanical stimulus-related genes were extracted from the databases, and then the least absolute shrinkage and selection operator (LASSO) regression analysis was used to construct a risk model. A nomogram integrating the genes and the clinicopathological characteristics was established and validated using the Kaplan-Meier survival and receiver operating characteristic (ROC) curves to estimate the overall survival (OS) of EC patients. Protein profiling technology and immunofluorescence technique were performed to verify the connection between biomechanics and EC.

**Results:**

In total, 79 mechanical stimulus-related genes were identified by analyzing the two databases. Based on the LASSO regression analysis, 7 genes were selected for the establishment of the risk model. This model showed a good performance in terms of the prognostic accuracy in high- and low-risk groups. The area under the ROC curves (AUC) of this model was 0.697, 0.712 and 0.723 for 3-, 5- and 7-year OS, respectively. Then, a nomogram integrating the genes of the risk model and clinical features was constructed. The nomogram could accurately predict the OS (AUC = 0.779, 0.812 and 0.806 for 3-, 5- and 7-year OS, respectively). The results of the protein profiling technology and immunofluorescence revealed the expression of cytoskeleton proteins to be correlated with the Matrigel stiffness degree.

**Conclusions:**

In summary, a risk model of 7 mechanical stimulus-related genes was identified in EC. A nomogram based on this risk model and combining the clinicopathological features to assess the overall survival of EC showed high practical value.

## Introduction

Endometrial cancer (EC) represents one of the most common gynecologic malignancies in China and around the world with an increasing incidence (1). About 75% of the EC cases are diagnosed at early stage (stage I or II), and the 5-year overall survival rate at stage III and IV in those with lymph node metastasis ranges from 20% to 71% (2). However, due to the unclear pathogenesis and molecular mechanism, the prognosis and management of EC are complicated. There is a need to explore the molecular mechanism of EC and detect the novel factors related to its diagnosis and prognosis.

Malignant cells undergo cell mechanics and particular biomechanical properties in their growth, invasion and extravasation (3). Therefore, biomechanics play a key role in the pathogenesis, diagnosis and prognosis of malignant tumors. However, current studies focus more on the cellular mechanism, while the clinical level is rarely involved.

In this study, we identified a set of EC genes that are related to the mechanical stimulus according to The Cancer Genome Atlas (TCGA) and Molecular Signatures Database (MSigDB). After comprehensive analysis of both TCGA and MSigDB databases, we found 79 mechanical stimulus-related genes from the common differentially expressed genes (DEGs) and constructed a risk model using the least absolute shrinkage and selection operator (LASSO) analysis. Afterwards, we established a nomogram combining the integrated clinicopathological features and mechanical stimulus-related risk signature to predict the overall survival (OS). We further explored the 7 key mechanical stimulus-related genes regarding their different expression and independent prognosis role. Meanwhile, the results of the protein profile immunofluorescence technique verified the interaction between the biomechanics and EC.

## Methods and Materials

### Data Acquisition and the Identification of Differentially Expressed Genes

We retrieved the data of the mRNA sequences and corresponding clinicopathological characteristics of 531 uterine corpus endometrial carcinoma (UCEC) tissues and 35 normal tissues from the TCGA (https://portal.gdc.cancer.gov/) database. In addition, 208 genes associated with the response to mechanical stimulus (GO:0009612) were obtained from the Molecular Signatures Database (MSigDB http://www.gsea-msigdb.org/gsea/index.jsp). We analyzed the mechanical stimulus-related DEGs between EC and the normal tissues using the limma package in the R software, which uses the significance analysis of the microarray method, following the criteria of log_2_|FC| > 1 and *P*-value < 0.05. The heatmap was plotted for the samples and DEGs were identified with the heatmap package in the R software. These overlapping genes were chosen for further analysis. Since the data were retrieved from open resource databases, no ethical approval was needed.

### Functional Enrichment Analysis of the Intersection Genes

In this study, to investigate the underlying mechanism of the DEGs, 79 DEGs were analyzed using the following tools. The clusterProfiler Bioconductor package was used to conduct GO pathway enrichment analysis of the overlapping DEGs. Disease ontology (DO) annotates the genes based on human diseases. DO analysis was performed to reveal the clinical relevance of the obtained key genes. Then, the DOSE package of the R software was used to analyze the semantic similarity computations of the DO terms and genes, thus revealing the closeness between diseases and gene functions. A *P*-value < 0.05 was set as the threshold value.

### Establishment and Verification of the Risk Model

The LASSO regression analysis was performed to narrow the range of the DEGs and establish a predicted model using the glmnet R package. The LASSO regression model was used to identify the most accurate predictive genes. Next, DEGs were filtered more than 1000 times. DEGs that were discovered more than 800 times in the LASSO analysis were selected as candidate prognostic genes, and the secondary related genes were filtered out.

Then, the mechanical stimulus-related hub genes selected using the LASSO analysis were used to construct a risk signature based on the coefficient for each patient according to the following formula:


$$RS=\sum_{i=1}^{n}Coef(i)X(i)$$

where Coef (i) denotes the coefficient, and X(i) is the z-score transformed relative expression level for each DEG. The prognostic risk score of each patient was calculated using the formula, and according to the median score, the patients were classified into high- and low-risk groups. Then, time-dependent receiver operating characteristic (ROC) curve (tdROC) and Kaplan-Meier survival curve analyses were performed to verify this risk score system. The pheatmap R package was used to compare the expression of the hub genes between high- and low-risk EC patients with different clinicopathological characteristics.

### Development and Validation of the Nomogram Based on the Risk Signature

Univariate and multivariate Cox regression analyses were carried out to evaluate the independent risk factors for OS, combined with the risk score and significant clinical signature. Then, we constructed the nomogram using these independent risk factors by the rms R package. The nomogram calculated the prognosis risk value for each patient and classified them into low-, moderate- and high-score groups. Furthermore, calibration curves were drafted and the concordance index (C-index) was calculated to assess the accuracy of the nomogram. The Kaplan-Meier curves drawn using the survival and survival ROC R packages were used to further confirm the predictive specificity of the risk model, *via* comparing the area under the tdROC curve (AUC) in different clinicopathological subgroups (such as the age, histological type and lymph node metastasis). A *P*-value < 0.05 was considered to be statistically significant.

### Verifying the Relationship Between Biomechanics and Endometrial Cancer Cell Line

In order to verify the connection between the mechanical force and endometrial cancer cells, we seeded Ishikawa cells on polyacrylamide hydrogel with different stiffness degrees (500pa, 10kpa and 87kpa). Then, we used protein profiling technology to detect the DEGs in these groups, and GO and Kyoto Encyclopedia for Genes and Genomes (KEGG) analyses were utilized to discover the enriched pathways of the DEGs. The immunofluorescence technique was conducted to observe the different expression of cytoskeleton protein (F-actin and paxillin) on hydrogel with different stiffness degrees. A *P*-value < 0.05 was considered to be statistically significant.

### Cell Culture

The EC cell line Ishikawa were obtained from laboratory stocks in Peking University People’s Hospital and cultured at 5% CO_2_ and 37°C in DMED/F12 medium (Gibco, Invitrogen, Carlsbad, CA, USA) supplied with 10% fetal bovine serum (Gibco, Invitrogen). Cell were subcultured every 2 days for cell experiments.

### Statistical Analysis

Continuous variables were expressed as mean ± SD (standard deviation) or median, while categorized variables were described by the frequency (n) and proportion (%). Differences among variables were tested using the Student’s t-test, chi-square test, one-way ANOVA tests or nonparametric tests. The log-rank test was applied to figure out the difference in the overall survival rate between the high-risk and low-risk groups. Statistical analyses were performed using the GraphPad prism 8 and R software, version 3.5.1. For all the analyses, differences were considered to be statistically significant if the *P*-values were less than 0.05.

## Results

### Identification of Mechanical Stimulus-Related Differentially Expressed Genes and Functional Analysis

**Figure 1:**
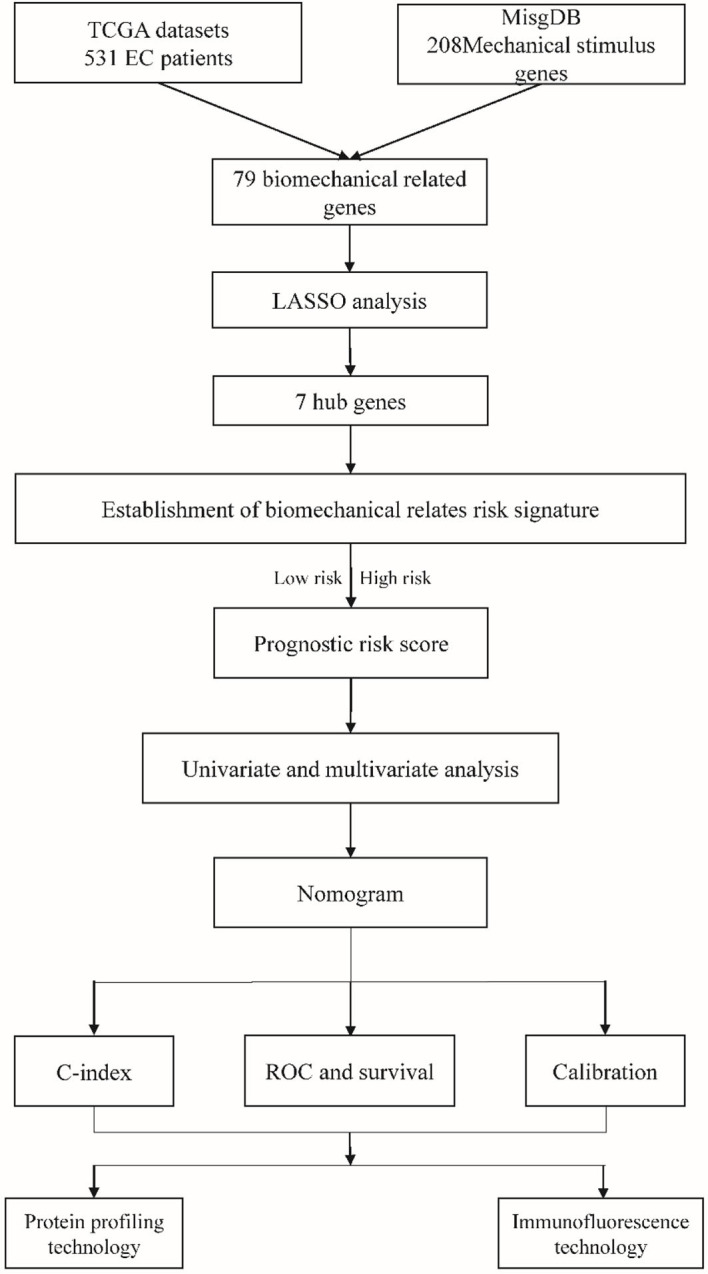
The flow chart of this study.

A total of 531 endometrial cancer patients were retrieved from the TCGA database and 208 mechanical stimulus-related genes were obtained from the MSigDB, these data were analyzed as shown in the flowchart (**Figure 1**).

**Figure 2:**
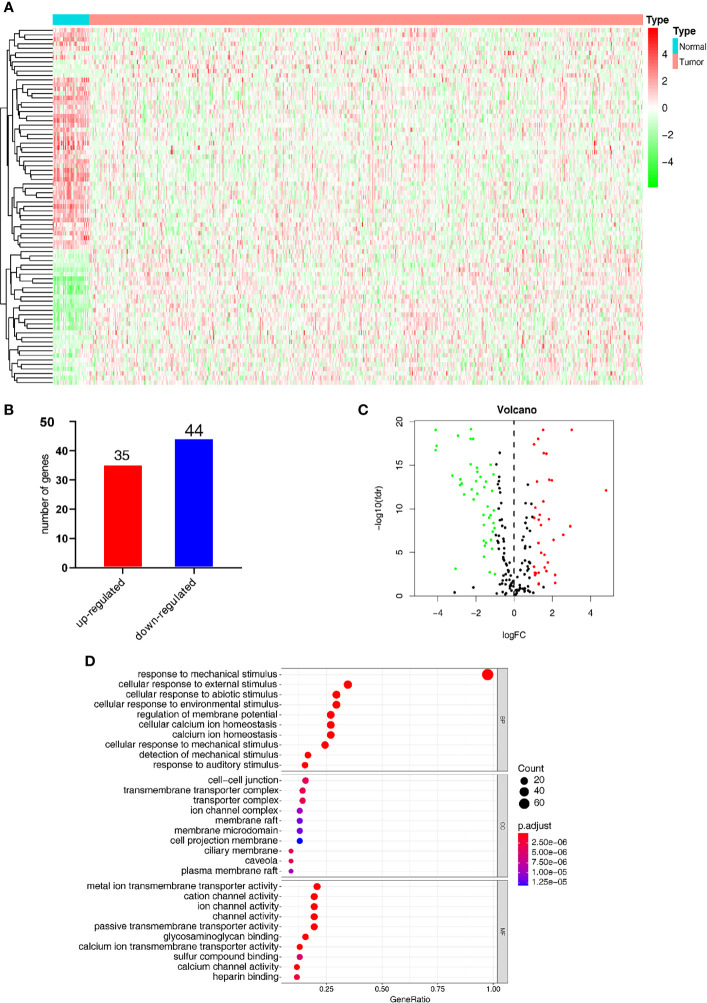
Identification of the mechanical stimulus-related key genes. **(A)** Heatmap of the expression of DEGs from the TCGA database. **(B)** The overlapped DEGs collected from both TCGA and GO databases. **(C)** The volcano plot of the mechanical stimulus-related genes in the two databases. **(D)** GO analysis of these DEGs.

**Figure 2A** shows the heatmap of the expression of the DEGs in tumor tissues and normal tissues collected from the TCGA database. In the MSigDB, 208 mechanical stimulus-related genes were found in oncology tissues. There were 79 overlapping DEGs between the TCGA database and MSigDB. Among the DEGs between tumor tissues and normal tissues, 35 genes were upregulated and 44 were downregulated (**Figure 2B**). The volcano plot shows the mechanical stimulus-related DEGs in the datasets (**Figure 2C**).

GO analysis was carried out to elucidate the potential function of these 79 overlapping genes. As shown in **Figure 2D**, these DEGs were mostly significantly enriched in the biological process (BP) term of “response to mechanical stimulus” (*P*-value < 0.05), in addition to the cellular component (CC) term of “cellular response to external stimulus” and molecular function (MF) term of “cellular response to abiotic stimulus. These results indicated that these DEGs were distributed in the mechanical stimulus-related processes and pathways, which have been proved to play a pivotal role in the tumorigenesis and progression of malignant tumors.

### Mechanical Stimulus-Related Risk Model Construction of the DEGs

**Figure 3:**
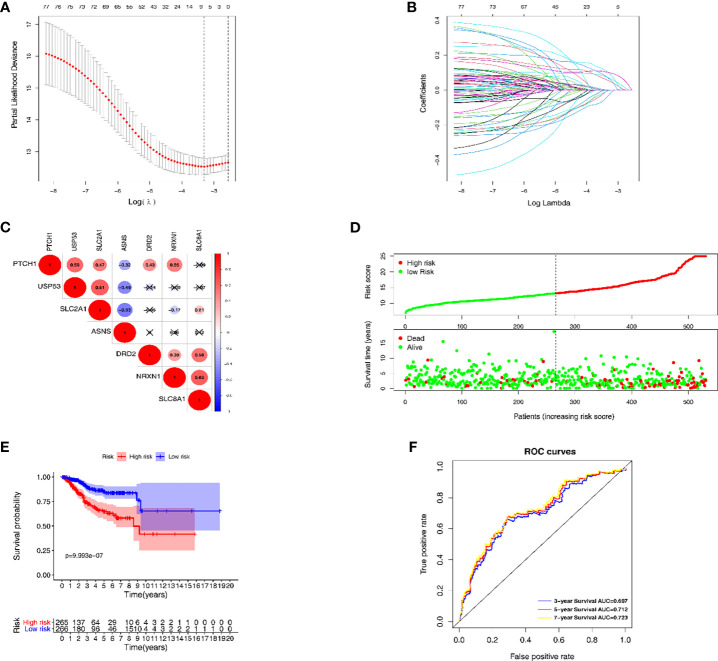
The establishment and verification of the LASSO regression model. **(A, B)** the evaluation progress of gene selection in LASSO regression. **(C)** The interrelation of 7 key DEGs acquired from LASSO regression. **(D)** High- and low-risk groups according to the risk score. **(E)** Kaplan-Meier survival analysis of the high- and low-risk groups. **(F)** Time dependent ROC curves for 3-year, 5-year and 7-year survival prediction.

The LASSO regression model was conducted using the expression profiles data of 79 DEGs to establish a mechanical stimulus-related risk model for the prognosis of EC. We performed 6-fold cross-validation and 7 genes were finally found with regression coefficients including *ASNS*, *DRD2*, *NRXN1*, *PTCH1*, *SLC2A1*, *SLC8A1* and *USP5* (**Figures 3A, B** and **Table 1**). The expression levels of the 7 genes were significantly correlated with each other, especially between *USP53* and *PTCH1*, *USP53* and *SLC2A1*, *NRXN1* and *SLC8A1* (**Figure 3C**). The risk score formulation for this risk signature was established as follows: risk score = (0.06826*ASNS) + (0.05467*DRD2) + (0.07154*NRXN1) + (0.02918*PTCH1) + (0.10020*SLC2A1) + (0.10164*SLC8A1) + (-0.00169* USP53). Then, each patient was evaluated according to the risk score, and clustered into high- and low-risk groups to calculate the prognosis value of the risk score model (**Figure 3D**). The Kaplan-Meier survival curve showed that the survival of the low-risk group was obviously better than the high-risk group (**Figure 3E**). The AUC of the 7-gene risk model was 0.697 for the 3-year survival, 0.712 for the 5-year survival and 0.723 for the 7-year survival (**Figure 3F**).

**Table 1:** Mechanical stimulus-related genes and correlated coefficient value.

Gene	Coefficient
ASNS	0.06825528
DRD2	0.054669638
NRXN1	0.07153678
PTCH1	-0.029178017
SLC2A1	0.100200637
SLC8A1	0.10164386
USP53	-0.001690605

### Further Analysis of Key Genes With Clinicopathological Signatures

**Figure 4:**
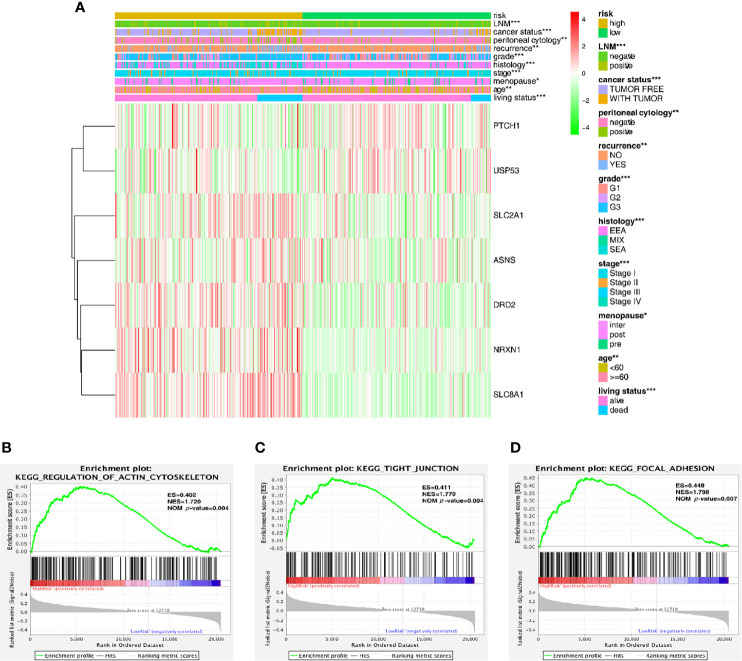
Predictive accuracy in high- and low-risk groups with differential clinicopathological signatures. **(A)** Heatmap with clinicopathological features in the two groups. Chi-square test was used for the correlation between clinical significance and the cluster. *p < 0.05, **p < 0.01 and ***p < 0.001. **(B–D)** GSEA analysis showing that the higher expression genes in the high-risk group are enriched in the regulation of actin cytoskeleton, tight junction and focal adhesion.

The expression level of the 7 key genes in high- and low-risk patients retrieved from the TCGA database with the clinicopathological signatures were presented in the heatmap (**Figure 4A** and **Table 2**). The results revealed significant differences between the low- and high-risk groups in terms of the lymph node metastasis (LNM) (*P*-value < 0.001), cancer status (*P*-value < 0.001), peritoneal cytology (*P*-value < 0.01), recurrence (*P*-value < 0.01), grade (*P*-value < 0.001), histology (*P*-value < 0.001), stage (*P*-value < 0.001), age (*P*-value < 0.01) and living status (*P*-value < 0.001).

**Table 2:** Clinicopathological signatures of patients in high- and low-risk.

Variables	Whole	Low risk	High risk	*p*-value
Total number	531	266	265	
Age (year)	N (%)			<0.01
<60	177 (33.33)	115 (43.23)	62 (23.40)	
>=60	354 (66.67)	151 (56.77)	203 (76.60)	
Living status				<0.001
Alive	439 (82.67)	238 (89.47)	201 (75.85)	
Dead	92 (17.33)	28 (10.53)	64 (24.15)	
Cancer status				<0.001
Tumor free	428 (80.60)	235 (88.35)	193 (72.83)	
With tumor	103 (19.40)	31 (11.65)	72 (27.17)	
Menopausal status				<0.05
Menstruate	16 (3.01)	13 (4.89)	3 (1.13)	
Premenopausal	58 (10.92)	34 (12.78)	24 (9.06)	
Postmenopausal	457 (86.06)	219 (82.33)	238 (89.81)	
FIGO stage				<0.001
Stage I	333 (62.71)	195 (73.31)	138 (52.08)	
Stage II	52 (9.79)	22 (8.27)	30 (11.32)	
Stage III	118 (22.22)	41 (15.47)	77 (29.06)	
Stage IV	28 (5.27)	8 (3.01)	20 (7.55)	
Tumor grade				<0.001
G1	98 (18.46)	64 (24.06)	34 (12.83)	
G2	118 (22.22)	80 (30.08)	38 (14.34)	
G3	315 (59.32)	122 (45.86)	193 (72.83)	
Histological type				<0.001
EEA	398 (74.95)	247 (92.86)	151 (56.98)	
Other types	133 (25.05)	19 (7.14)	114 (43.02)	
Recurrence				<0.01
No	426 (80.23)	229 (86.09)	197 (74.34)	
Yes	105 (19.77)	37 (13.91)	68 (25.66)	
Peritoneal cytology				<0.01
Negative	457 (86.06)	245 (92.11)	212 (80.00)	
Positive	74 (13.94)	21 (7.89)	53 (20.00)	
LNM				<0.001
Negative	450 (84.75)	240 (90.23)	210 (79.25)	
Positive	81 (15.25)	26 (9.77)	55 (20.75)	

EEA, endometrioid endometrial adenocarcinoma; FIGO, international federation of gynecology and obstetrics; LNM, lymph node metastasis.

Furthermore, the gene set enrichment analysis (GSEA) was conducted to investigate the role of these key genes in the endometrial cancer tumorigenesis mechanism. As shown in **Figures 4B–D**, these genes might play a crucial role in the regulation of actin cytoskeleton (ES = 0.402, NOM *P*-value = 0.004), tight junction (ES = 0.411, NOM *P*-value = 0.004) and focal adhesion (ES = 0.448, NOM *P*-value = 0.007); all of these cell signal pathways were related to the mechanical stimulus. After all, the risk group built on the mechanical stimulus-related signature of 7 key genes might be distinguishable when the EC patients were classified according to our high- and low-risk models, and the mechanism might be involved in the pathway responding to mechanical stimulus.

### Establishment and Evaluation of a Prognostic Nomogram

**Figure 5:**
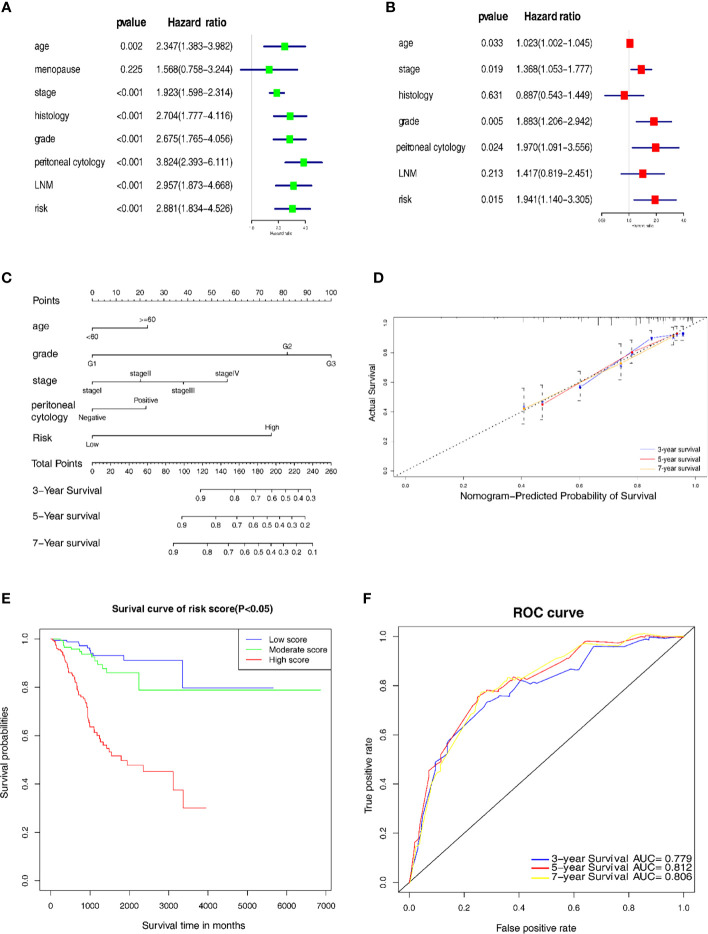
Establishment of the nomogram for prognostic prediction. **(A)** Univariate analyses of the risk model with clinicopathological features. **(B)** Multivariate analysis of the risk model with clinicopathological features. **(C)** Nomogram including the 7 key genes, risk score and clinicopathological signatures. **(D)** Calibration plot of the nomogram prediction possibility. **(E)** Kaplan-Meier survival analysis of the patients in the risk score. **(F)** Time-dependent ROC curve in the risk model.

Univariate and multivariate Cox regression analyses were used to distinguish the independent risk factors by developing a nomogram used to further predict the clinical outcome of EC patients with the mechanical stimulus-related risk model. **Figure 5A** showed the evaluation of the univariate analysis, and **Figure 5B** showed the result of multivariate analysis, suggesting that the age (Hazard ratio(HR) = 1.023, 95% Confidence Interval (CI): 1.002-1.045, *P*-value = 0.033), stage (HR = 1.368, 95%CI: 1.053-1.777, *P*-value = 0.019), grade (HR = 1.883 95%CI: 1.206−2.942, *P*-value = 0.005), peritoneal cytology (HR = 1.970, 95%CI: 1.091-3.556, *P*-value = 0.024) and risk signature (HR = 1.941, 95%CI: 1.140−3.305, *P*-value = 0.015) were found to be independent risk factors for the overall survival of EC patients. Then, we designed a nomogram to predict the probability of OS (**Figure 5C**). In the nomogram, each predictor was assigned a score, based on the multivariate analysis results, and a total of 5 factors were integrated in the nomogram to predict the OS of EC patients. The calibration curve suggested that the nomogram that predicted the survival rate was close to the actual situation for all of the 3-, 5-, and 7-year survival (**Figure 5D**).

In addition, we categorized the patients into 3 subgroups according to their total points and further tested the survival assessment model using the Kaplan-Meier analysis in both the whole cohort and subgroups. The results showed that this nomogram could perform well in partitioning the patients in the whole groups (**Figure 5E**). The AUC of the nomogram was 0.779, 0.812 and 0.806 for the 3-, 5- and 7-year OS, respectively (**Figure 5F**). These findings revealed that this risk signature-based nomogram had definite reliability and specificity in evaluating the prognosis.

### Further Verification of the Nomogram in Different Clinicopathological Subgroups

**Figure 6:**
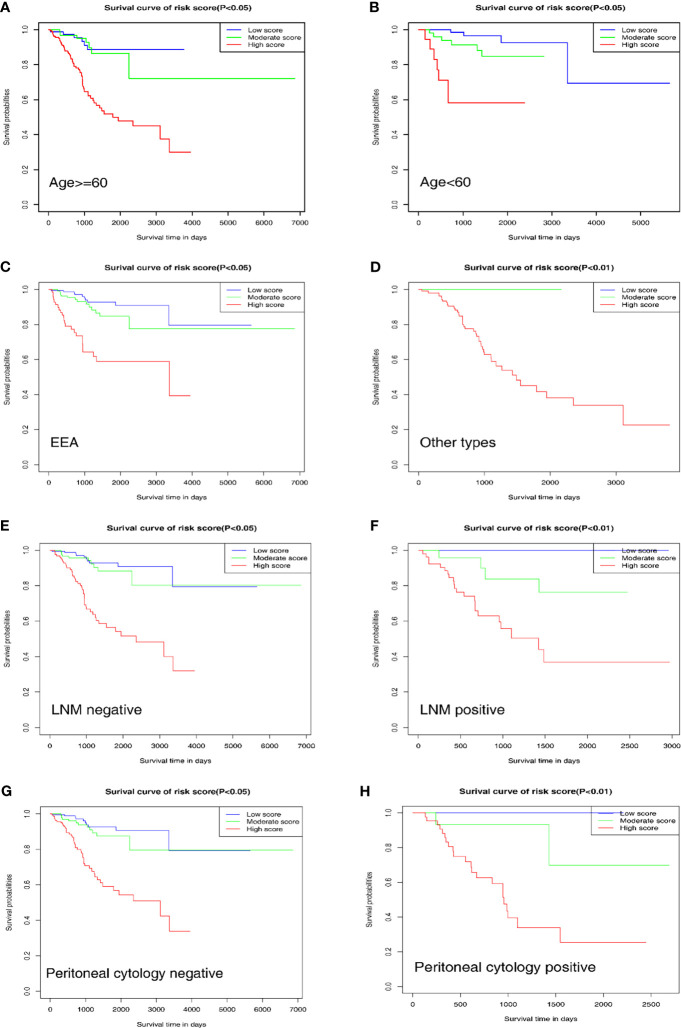
Prognostic value of the nomogram in different clinicopathological subgroups. **(A, B)** Age subgroups. **(C, D)** Histological type subgroups. **(E, F)** Lymph node metastasis subgroups. **(G, H)** Peritoneal cytology subgroups.

To further evaluate and verify the survival assessment model, we conducted the Kaplan–Meier survival analysis in the following different clinicopathological subgroups of the patients: age subgroups, age < 60 and age ≥ 60; histological type subgroups, EEA and other types; lymph node metastasis subgroups, negative and positive; peritoneal cytology subgroups, negative and positive. The tests showed that the predictive capability of the survival assessment model was effective in all the clinicopathological subgroups of EC patients (**Figures 6A–H**). Thus, this mechanical stimulus-related risk model had a certain reliability and practicability in evaluating the prognosis.

### Excavation of the Relationship Between Biomechanical Genes and the Pathogenesis of EC

**Figure 7:**
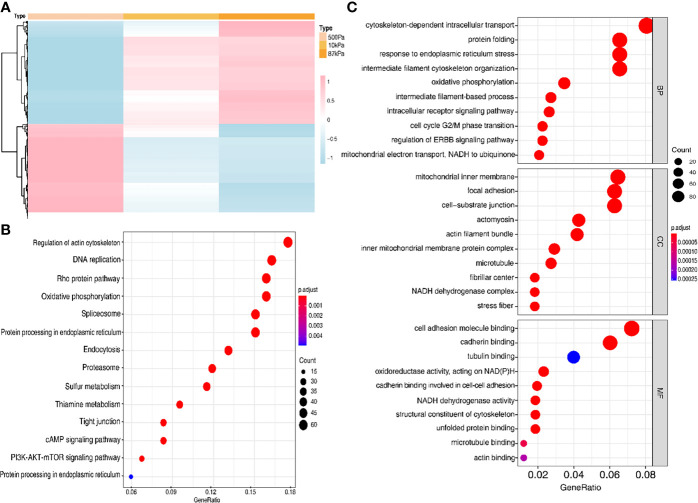
The protein expression of the Ishikawa cells on polyacrylamide hydrogel with different stiffness degrees. **(A)** Heatmap of the proteins with different expression levels in hydrogel. **(B, C)** GO analysis of the proteins with different expression levels.

In order to verify the prediction results, we conducted experiments to explore the effects of different mechanical stimulations on the function of EC cells. The results of the protein profiling technology in different stiffness degrees (500kPa, 10kPa and 87kpa) indicated a clearly different expression level of the proteins in these groups (**Figure 7A**).

**Figure 8:**
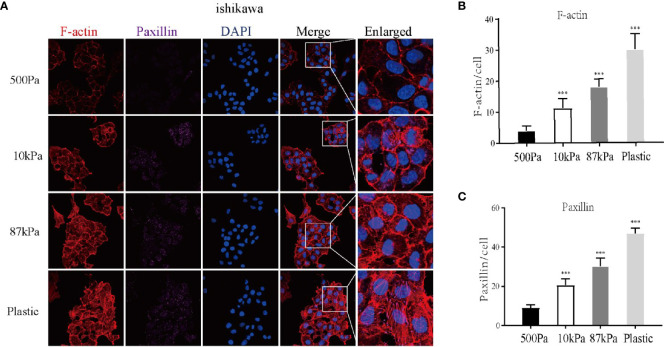
Expression of the Ishikawa cytoskeleton proteins on Matrigel with different stiffness degrees. **(A)** The imagines of F-actin and paxillin. **(B, C )** The expression of F-actin, paxillin and pseudopodia in Ishikawa cells. **p < 0.01 and ***p < 0.001.

Moreover, GO analysis demonstrated that the function of these proteins obviously concentrated on the regulation of actin cytoskeleton (**Figure 7B**) and the enriched bp term was the “cytoskeleton-dependent intracellular transport”, cc term was the “mitochondrial inner membrane” and mf term was the “cell adhesion molecule binding” (**Figure 7C**). Furthermore, the Ishikawa cells seeded in the three groups showed a significant difference in the cytoskeleton proteins (F-actin and Paxillin), with the increase in stiffness, the expression of cytoskeleton protein was strengthened. Meanwhile, the number of pseudopodia growing with the increase in stiffness indicated the enhancement of cell motility (**Figure 8**). These results demonstrated that biomechanical genes might play a crucial role in the malignant process of EC, especially in metastasis and invasion.

## Discussion

Endometrial cancer is one of the most common gynecologic malignant tumors with an increasing incidence of high-grade, clinically aggressive tumors (4). At the advanced stage, the rate of 5-year overall survival approximately reaches 15% (5). Therefore, it is of great significance to discover the diagnosis and prognosis biomarkers and to seek out the mechanism behind the metastasis and invasion of EC. Nowadays, bioinformatics analysis is widely used to explore the crucial genes in the pathogenesis and prognosis of various types of tumors. Besides, the nomogram model is being more used to predict the prognosis of cancer using the clinicopathological signatures. A previous study (6) used bioinformatics analysis and discovered that the FOXL2, TCF4 and NR2F2 transcription factors could serve as biomarkers in the prognosis and potential targets in clinical therapy. Yet, biomarkers explored by most of the previous studies focused on the molecular factors. In our study, clinical and molecular data combined with the genomic characteristics were explored to develop the prognostic model and improve the accuracy.

In the progress of cancer growth, metastasis, invasion and adhesion, malignant cells demand particular mechanical properties (6). Tumor biomechanics may regulate the cytoskeleton signaling pathway to induct the malignant behavior. A previous study expounded that Talin2 may bind to β-integrin to regulate the tumor cell migration and invasion, playing a key role in cancer cell invasion (7). In breast cancer, researchers also found that the remodeling and reorganization of cytoskeleton and cell-matrix could alter the traction forces, thereby modulating the cell motility behavior (8). However, the relationship between biomechanics and EC has rarely been investigated. In this study, we found that the mechanical stimulus-related genes could act as predictive factors in the prognosis of EC. Then, we verified their biological functions in the development of cancer. Our results indicated that these key genes were related to the cytoskeleton regulation.

The TCGA database was used to retrieve the genomic data of endometrial cancer patients, and 79 overlapped genes were found. Using the LASSO analysis, 7 key genes (*ASNS*, *DRD2*, *NRXN1*, *PTCH1*, *SLC2A1*, *SLC8A1* and *USP5*) were sought out to establish the prognosis model. *ASNS* is involved in the synthesis of asparagine, progression of the cell cycle and sensitivity to cisplatin of malignant tumors (9). *DRD2* encodes a kind of dopamine receptors and represents a tumor suppressor educating M1 macrophages by the NF-κB signaling pathway (10). *NRXN1* encodes a membrane protein that is interrelated with the transfer of Ca^2+^ (11). Previous studies demonstrated that *PTCH1* could induct the malignant cell stemness and facilitate the cell autophagy (12, 13). *SLC2A1* and *SLC8A1* both belong to the solute carrier family. *SLC2A1* encodes the glucose transporter, which is associated with the abnormal metabolism during cancer cell invasion (14), while *SLC8A1* participates in the migration of tumor cells *via* regulating Na^+^/Ca^2+^ exchange (15), it is also involved in the endometrial tissue cellular heterogeneity (16). Finally, *USP5* encodes a metabolism protein that is highly expressed in many kinds of cancer, such as breast, prostate and hepatic cancer (17). The results of our study show that the functions of these genes are enriched in the biomechanical signal pathways, including the regulation of actin cytoskeleton, tight junction and focal adhesion.

In addition, our study analyzed much data from the TCGA database and focused on the gene expression of mechanical stimulus-related genes. Compared with previous studies on EC prediction biomarkers, in our study, a nomogram was used as a graphical tool to evaluate the prediction factors more tangibly and intuitively interpret the predictors with the clinical signatures (18). Our nomogram displayed that the biomechanical genes could be independent prognostic predictors of EC, which was verified *via* the Kaplan-Meier analysis in the clinicopathological groups, especially in the age, histological type, lymph node metastasis and peritoneal cytology subgroups.

This study showed that mechanical stimulus played an important role in endometrial cancer prognosis. Moreover, the tumor cells were subjected to a variety of mechanical forces, and the stiffness of the extracellular matrix is one of the main forces (19). In order to further verify the relationship between mechanical factors and endometrial cancer, we used polyacrylamide hydrogels with different stiffness degrees to simulate the extracellular matrix with different stiffness, then the protein profiling technology analyzed the protein expression of Ishikawa cells seeded in polyacrylamide hydrogels with different stiffness degrees. Through GO analysis, we found these DEGs to be enriched in the pathway of the regulation of actin cytoskeleton. Cytoskeleton is crucial for cell deformation and shape changing, internal and external forces could affect the cell mechanical characteristics *via* regulating cytoskeleton (20). Besides, cytoskeleton is the inductor and presenter of cell migration and invasion in malignant tumors (21). Our group previously reported that transient receptor potential vanilloid 4(TRPV4) was involved in EC cells migration through cytoskeletal regulation (22). We further demonstrated the expression of cytoskeleton with different stiffness degrees of hydrogels by cellular experiments, results showed that with the increase in stiffness, the expression of cytoskeleton protein was enhanced and the number of pseudopodia was increased. This result illustrated that the increasing stiffness of hydrogels could promote the motility of EC cells by regulating cytoskeleton.

Taken together, our study identified 7 mechanical stimulus-related genes *via* the LASSO analysis and set up a nomogram predictive model for EC patients. In the validation group, the OS of the high-risk group associated with the age, lymph node metastasis, ascites metastasis and FIGO stage were obviously worse than the patients in the low-risk group. Our study showed that cellular mechanics represented a considerable factor participating in the progression and invasion of EC.

## Conclusion

In summary, we constructed a prognostic risk model with 7 mechanical stimulus-related genes and established a prognostic prediction tool in the form of an integrative nomogram that could accurately and specifically forecast the clinical outcome of EC. We also explored the functions EC cells on hydrogels with different stiffness degrees. Although the clear mechanism of biomechanics regulating the progress of EC remains in need for further exploration, this study contributes to promoting the research on the mechanisms of tumor malignant behavior in EC.

## Data Availability Statement

The raw data supporting the conclusions of this article will be made available by the authors, without undue reservation.

## Author Contributions

Writing-Original Draft Preparation, XX. Data Curation, XL. Writing-Review and Editing, JZ and JW. All authors contributed to the article and approved the submitted version.

## Funding

This study is supported by the National Key Technology R&D Program of China (Grant Number. 2019YFC1005200 and 2019YFC1005201), Peking University Medicine Fund of Fostering Young Scholars’ Scientific & Technological Innovation (Grant Number. BMU2021PYB012 and BMU2021MX006) and National Natural Science Foundation of China (Grant Number. 81874108 and 82072861).

## Conflict of Interest

The authors declare that the research was conducted in the absence of any commercial or financial relationships that could be construed as a potential conflict of interest.

## Publisher’s Note

All claims expressed in this article are solely those of the authors and do not necessarily represent those of their affiliated organizations, or those of the publisher, the editors and the reviewers. Any product that may be evaluated in this article, or claim that may be made by its manufacturer, is not guaranteed or endorsed by the publisher.

## Abbreviations

EC, endometrial cancer; TCGA, The cancer genome atlas; MSigDB, Molecular Signatures Database; LASSO, least absolute shrinkage and selection operator; ROC, receiver operating characteristic; tdROC, time-dependent receiver operating characteristic; AUC, area under the ROC curve; C-index, concordance index; UCEC, uterine corpus endometrial carcinoma; DEGs, Differentially expressed genes; HR, hazard ratio; ES, enrichment score; NES, normalized enrichment score; EEA, endometrioid endometrial adenocarcinoma; SEA, serous endometrial adenocarcinoma; FIGO, international federation of gynecology and obstetrics; G1/2/3, grade 1/2/3; LNM, lymph node metastasis; OS, overall survival.
